# Working Memory And Brain Tissue Microstructure: White Matter Tract Integrity Based On Multi-Shell Diffusion MRI

**DOI:** 10.1038/s41598-018-21428-4

**Published:** 2018-02-16

**Authors:** Sohae Chung, Els Fieremans, Nuri E. Kucukboyaci, Xiuyuan Wang, Charles J. Morton, Dmitry S. Novikov, Joseph F. Rath, Yvonne W. Lui

**Affiliations:** 1 Department of Radiology, Center for Advanced Imaging Innovation and Research (CAI2R), New York University School of Medicine, New York, NY 10016 USA; 2 Department of Radiology, Bernard and Irene Schwartz Center for Biomedical Imaging, New York University School of Medicine, New York, NY 10016 USA; 3 Kessler Foundation, East Hanover, NJ 07936 USA; 4 Department of Rehabilitation Medicine, New York University School of Medicine, New York, NY 10016 USA

## Abstract

Working memory is a complex cognitive process at the intersection of sensory processing, learning, and short-term memory and also has a general executive attention component. Impaired working memory is associated with a range of neurological and psychiatric disorders, but very little is known about how working memory relates to underlying white matter (WM) microstructure. In this study, we investigate the association between WM microstructure and performance on working memory tasks in healthy adults (right-handed, native English speakers). We combine compartment specific WM tract integrity (WMTI) metrics derived from multi-shell diffusion MRI as well as diffusion tensor/kurtosis imaging (DTI/DKI) metrics with Wechsler Adult Intelligence Scale-Fourth Edition (WAIS-IV) subtests tapping auditory working memory. WMTI is a novel tool that helps us describe the microstructural characteristics in both the intra- and extra-axonal environments of WM such as axonal water fraction (AWF), intra-axonal diffusivity, extra-axonal axial and radial diffusivities, allowing a more biophysical interpretation of WM changes. We demonstrate significant positive correlations between AWF and letter-number sequencing (LNS), suggesting that higher AWF with better performance on complex, more demanding auditory working memory tasks goes along with greater axonal volume and greater myelination in specific regions, causing efficient and faster information process.

## Introduction

Working memory is traditionally conceptualized as a hierarchical system with limited capacity and duration at the core of cognition and consciousness. It is composed of three main components: (1) the phonological loop which provides short-term memory traces for sounds by continuously refreshing the information through silent rehearsal; (2) the visual-spatial sketchpad which allows individuals to momentarily create and revisit a mental image that can be manipulated in complex tasks; and (3) the central executive which involves selective attention, inhibition, and shifting between tasks^1–4^. Involving both maintenance and manipulation of information, working memory is essential for higher-order functions such as comprehension, learning, reasoning, and decision making^2,5^. Deficits in working memory are fundamental problems associated with a wide range of progressive and non-progressive conditions including developmental disorders, learning disabilities, traumatic brain injury, stroke, and multiple sclerosis^6–9^. In fact, macrostructural alterations of the brain including focal decreases in brain volume^10,11^, cortical thickness^12^, and hippocampal volume^13^ have previously been associated with cognitive disability.

There is specific interest in understanding the microstructural correlates that contribute to such macroscopic changes as well as inform more subtle differences in working memory performance. Several diffusion tensor imaging (DTI) studies of white matter (WM) reveal positive correlations between fractional anisotropy (FA) and performance of a working memory task in fronto-parietal WM in children^14–16^ and adults^17^; and also in the corpus callosum and posterior temporal WM in younger children^18^. Positive correlations between FA and verbal working memory performance have also been reported in right precuneal WM in normal young adults^19^. FA is, however, a nonspecific measure of directional diffusion, affected by a number of biophysical factors such as extracellular water, myelination, axon thickness and density^20^. In addition, diffusion kurtosis imaging (DKI)^21^ has been employed to characterize non-Gaussian diffusion and studies show an association between mean kurtosis (MK), a reflection of tissue microstructural complexity, and cognitive deficits in a number of pathologies including multiple sclerosis^22^, mild cognitive impairment^23^, Alzheimer’s^23^, and mild traumatic brain injury^24^. FA and MK, being empirical measures, intrinsically lack specific biophysical meaning. Thus, it remains unclear what biological structural differences may underlie the observed diffusion signal changes.

Most recently, compartment-specific WM tract integrity (WMTI) metrics derived from a WM modeling of multi-shell diffusion magnetic resonance imaging (MRI) have the potential to disentangle intra- and extra-axonal environments^25^. WMTI metrics include: 1) axonal water fraction [AWF], 2) intra-axonal diffusivity [${\text{D}}_{\text{axon}}$, diffusivity within axons], 3) extra-axonal axial and 4) extra-axonal radial diffusivities [${\text{D}}_{\text{e,}\Vert}$ and ${\text{D}}_{\text{e,}\bot}$, diffusion parallel and perpendicular to the axonal tracts in the extra-axonal space, respectively]. There are promising works showing WMTI metrics to be more specific to underlying tissue microstructure and mechanisms than empirical diffusion measures such as FA: WMTI metrics and tissue microstructure have been studied in several animal validation works, specifically examining demyelination and remyelination^26–29^, as well as in human *in vivo* studies of neural development and disease. Briefly, alterations in AWF and ${\text{D}}_{\text{e,}\bot}$ appear to reflect axon density and myelination both in normal development^30^ and in Alzheimer’s disease^31,32^; alterations in ${\text{D}}_{\text{axon}}$ have been shown in conditions of axon injury including stroke^33^ and mild traumatic brain injury^34^; and ${\text{D}}_{\text{e,}\Vert}$ and ${\text{D}}_{\text{e,}\bot}$ demonstrate sensitivity to changes in extra-axonal diffusion (such as demyelination, gliosis and astrocytosis, extracellular inflammation) in patients with mild cognitive impairment^31^. Specifically with regard to working memory and brain microstructure, prior reports show strong association of visual working memory with AWF in healthy subjects^35,36^.

Here we investigate the relationship between diffusion metrics and performance on Wechsler Adult Intelligence Scale-Fourth Edition (WAIS-IV)^37^ subtests tapping auditory working memory, including digit span forward (DSF), backward (DSB), sequencing (DSS), and letter-number sequencing (LNS) in healthy adults. The working memory tasks represent a progression from “simple span” tasks, focusing on short-term storage and deemphasizing the executive component of working memory, to “complex span” tasks which involve both storage and manipulation of information. In this progression, DSF, requiring simple repetition of numbers in the order presented, taps the phonological loop. DSB, which requires that numbers be re-ordered and repeated in reverse, taps both the phonological loop and executive components of working memory. DSS adds an element of semantic processing, because the meaning of the numbers presented must be comprehended in order to repeat them in ascending order. Finally, shifting between letters and numbers as required by LNS involves additional executive demands. LNS has been shown to involve processing speed and visual-spatial working memory components (relating to the strategy of visualizing numbers and letters as they are placed in ascending/alphabetical order) not tapped by DS tasks^38^. In order to avoid any confounding effects of language and handedness, we included only right-handed, native English speaking individuals in this study.

## Results

Length of education of our subjects varied between 12 and 20 years (16 ± 2 years) and the WRAT-4 IQ scores were ranged from 88 to 134 (111 ± 15). The WAIS-IV DS and LNS test scaled scores were ranged from 7 to 19 and age-corrected z-scores were ranged from −1 to 3. They were not significantly correlated with age and length of education, but we found a trend for DSS and LNS with length of education. These are summarized in Table 1.

**Table 1:** WAIS-IV working memory subtests scaled/z-scores (N = 15), and their correlation p-values with age and length of education.

	Scaled score			Z-score			Correlation with age (p-value)	Correlation with education (p-value)
	Mean ± SD	Min	Max	Mean ± SD	Min	Max		
DSF	11.73 ± 3.06	7	16	0.58 ± 1.02	−1	2	0.47	0.56
DSB	12.20 ± 2.65	9	18	0.67 ± 0.87	−0.33	2.67	0.60	0.15
DSS	11.67 ± 3.06	8	17	0.55 ± 1.02	−0.67	2.33	0.43	0.056
LNS	12.60 ± 3.33	9	19	0.87 ± 1.11	−0.33	3	0.44	0.053

**Figure 1:**
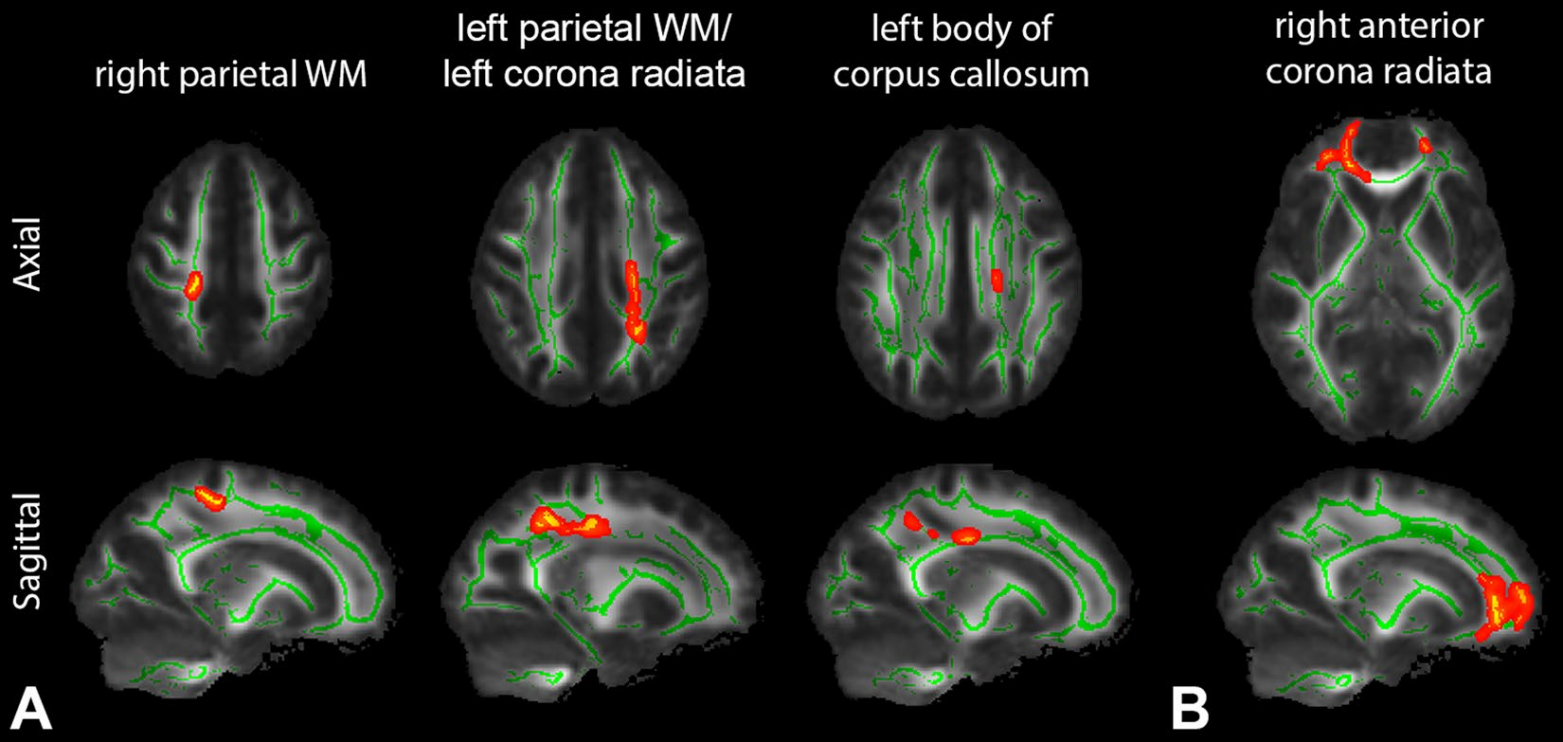
(**A**) Tract-based spatial statistics (TBSS) results showing significantly positive correlations between axonal water fraction (AWF) and WAIS-IV letter-number sequencing (LNS) test z-scores. Mean FA skeleton (green) overlaid on the mean FA map. Significantly correlated voxels (corrected p < 0.05) are shown in red-yellow and involve left greater than right parietal white matter (WM) (specifically based on the MNI atlas: right/left parietal WM, left superior and posterior corona radiata, left body of corpus callosum). (**B**) Significantly correlated voxels between mean kurtosis (MK) and LNS test z-scores are present in the right anterior corona radiata. No negative correlations were found. Identical results were found with WAIS-IV LNS test scaled scores.

From TBSS analysis, there were statistically significant positive correlations of AWF and MK with LNS at the 95% confidence level after multiple comparisons. As shown in Fig. 1A, this significant correlation between AWF and LNS was present mainly in parietal WM, more prominently on the left. Specific areas based on MNI atlas include: right/left parietal WM, left superior and posterior corona radiata, and left body of corpus callosum. A region in the right anterior corona radiata also showed a significant positive correlation between MK and LNS (Fig. 1B). No other diffusion metrics showed area of significant correlations surviving multiple comparison corrections. No significant correlations were found with performance on the DS tasks. We found essentially identical results between using scaled scores and using z-scores.

**Figure 2:**
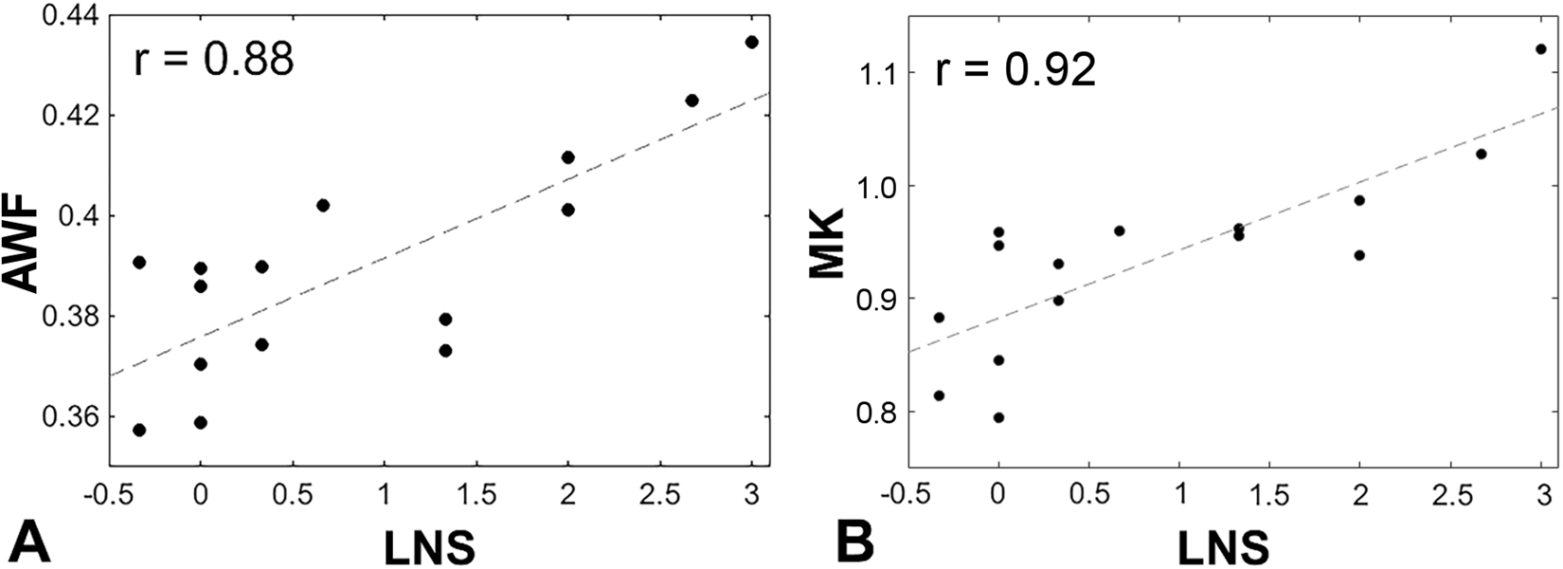
Scatter plots showing significant correlations. (**A**) Between AWF and LNS z-scores (r = 0.88) and (**B**) between MK and LNS z-scores (r = 0.92) for voxels on the skeleton with statistically significant association in TBSS (corrected p < 0.05; shown in Fig. 1).

Figure 2 presents the scatter plots of each significant metric and LNS z-score, showing that higher AWF (Fig. 2A; r = 0.88) and higher MK (Fig. 2B; r = 0.92) are associated with better performance on the LNS task for those significant voxels on the skeleton in TBSS shown in Fig. 1.

## Discussion

This study demonstrates statistically significant WM microstructural associations with performance on a select auditory working memory task, LNS. It is interesting to note that no significant correlations were observed with performance on the three relatively simpler WAIS-IV DS tasks (recalling numbers in order, backwards, and reordering in WAIS-IV DS subtest). However, when subjects were asked to perform LNS, the most complex and demanding WAIS-IV working memory subtest which requires shifting between letters and numbers, significant correlations were observed in AWF as well as MK. While processing speed has been shown to contribute to LNS performance^38^, past research agrees with the WAIS development team^37^ that many aspects of crystallized (e.g., high reading level^39^) and fluid intelligence (e.g., strong visuospatial working memory) may contribute to high scores in this test.

Our findings show WM microstructure to be more closely associated with more complex tasks of working memory that also require efficiencies in processing speed and fluid intelligence. The greater localization to the left hemisphere is consistent with typical lateralization for auditory-verbal working memory^40^. The correlation shown in a small right frontal region supports virtual visual rehearsal and manipulation that many individuals employ in order to successfully complete such tasks^38^. Indeed, a prior report shows associations of visual short-term memory with WM microstructure in the superior longitudinal fasciculus and inferior fronto-occipital fasciculus within the right hemisphere^41^. Our findings in the parietal WM regions may also relate to visual rehearsal^42^.

The correlation coefficients observed in the significant regions are high between AWF and LNS (r = 0.88), and between MK and LNS (r = 0.92). Higher AWF has previously been shown in two conditions; higher axonal density as well as greater myelination^28,43^. Higher MK reflects greater tissue microstructural complexity^29,44^. Differences between subjects in terms of axonal density and myelination would certainly be expected to influence neuronal signal transmission^45^ and could conceivably contribute to greater efficiency in information processing^38^.

We find no significant correlation between FA and these tests of working memory. The literature is variable with some papers showing positive correlations with FA though in differing brain regions^17,19^ and others showing no correlation^35,46^. This may be because FA is inherently a nonspecific empiric measure affected by multiple different microstructural features including myelination, axon density, extracellular fluid content, etc^47^.

This study includes subjects with age range of 19 to 45 years old. High average reliability of LNS scores (0.86–0.91) in our subjects across age and low (<14%) coefficient of variability^48^ seen here agree with prior research documenting relative stability of working memory performance in healthy adults, characterized by a slow decline in average scores beginning in middle age^49–51^. In addition, we address any remaining age effects by using age-corrected z-scores derived from a published normative sample (n = 2200) divided into 13 age bands, spanning ages 16 to 90^37^. This analysis was also cross-checked using the scaled scores of the cognitive tests, which showed no differences in the results. Also, DTI studies^52,53^ have revealed that this age range is a relatively stable period and possibly contributes to why age-related changes were not observed in this study. Furthermore, all statistical analyses included age and sex as covariates and were not found to have an effect on the findings.

TBSS, used here, is an established methodology, widely used for voxelwise analysis of whole brain WM diffusion measures. Limitations of TBSS include the fact that WM texture is not interrogated using this method as only the maximum value along a line orthogonal to the skeleton is projected onto the skeleton. Other potential problems that have been reported associated with TBSS including effects of partial volume, skeleton shapes, image noise level and registration error^54^. While TBSS certainly has limitations, TBSS has several strengths which make it an excellent choice of methodology including skeletonization which reduces the need for data smoothing, alleviates residual image misalignment and gains statistical power from reducing dimensionality^54^. In this study, default parameter settings have been employed and potentially problematic regions (e.g., low FA (<0.2) regions such as fornix, uncinate fasciculus) have not been included in the results, as recommended by Bach *et al*.^54^. By using skeletonized WM, TBSS is useful in substantially decreasing the number of comparisons compared with a voxel-by-voxel based approach; however, there remain potential limitations in the methodology applied to small sample size studies^55^. In particular, variance in diffusion metrics across healthy subjects has been reported, particularly affecting FA^55^. Variance in FA may reflect why no correlations in FA were detected in our study. Kurtosis measures, on the other hand, show less variance. Based on previously reported intersubject variance in DKI metrics, our study is powered to detect an effect size of 10% at 0.9 statistical power^56^. In particular, Jensen *et al*.^57^ have showed the WMTI model parameters to have deviations being less than 10% at FA threshold ≥0.3 which is much lower as detailed in the De Santis *et al*. paper^55^, putting the sample size employed in this work in a reasonable range. Moreover, potential error relating to default FA-based TBSS registration would be further improved by using a tensor-based registration^54^; however, it was not implemented in this study.

Finally, the WMTI model employed here makes two main assumptions regarding fiber alignment and diffusion compartmentalization: 1) It assumes regions of highly aligned fiber bundles and therefore, in this work, the WM regions were thresholded to FA ≥ 0.4 as has been previously described^25,57^ (about 30% of total WM) to restrict TBSS analysis to regions of reasonable high directional diffusion for calculation of WMTI metrics; FA threshold at 0.4 conservatively restricts the WM assessed to area where the WMTI model is valid based on Jensen *et al*.^57^, and 2) the model assumes ${\text{D}}_{\text{axon}}$ ≤ ${\text{D}}_{\text{e,}\Vert}$; which is supported by several animal validation studies^26,28^ though remains a topic of debate^58–61^; Nevertheless, AWF is independent of this latter assumption and therefore the main result of this investigation is not affected.

In summary, our findings support a real potential of diffusion metrics not only to identify WM microstructural associations with working memory but to begin to parse out the meaning behind such relationships, suggesting that higher AWF with better performance on LNS goes along with greater axonal volume and greater myelination in specific regions. Elucidating the links between brain microstructure and working memory has generalizable value across normal and pathologic conditions such as aging, dementia, and abnormalities of attention including attention deficit hyperactivity disorder (ADHD).

## Methods

### Study Population

This study was approved by the Institutional Review Board at New York University School of Medicine, and all experiments were performed in accordance with relevant guidelines and regulations. All subjects were prospectively recruited and provided written informed consent before the procedure. We studied 15 healthy individuals (mean age, 31 ± 7 years old; age range, 19–45 years old; 7 males). Exclusion criteria included: 1) reported history of brain disorders, head trauma or psychotic disorders; 2) non-native English speakers and 3) non-right-handed individuals to avoid confounding effects of language and handedness in both WAIS-IV test performance and WM microstructure. Per study procedures, all subjects underwent formal neurocognitive testing using WAIS-IV working memory subtests^37^ and a brain MRI scan within one day of each other. Wide Range Achievement Test-4^th^ Edition Word Reading subtest (WRAT-4) was also performed to help characterize subjects and the scores were converted to IQ scores for a brief measure of academic achievement.

### MRI Acquisition

MR imaging data were acquired on a 3 T MR scanner (Skyra, Siemens Medical Solutions, Erlangen, Germany). Diffusion imaging was performed at multiple shells: 5 b-values (250, 1000, 1500, 2000, 2500 s/mm^2^) along with 5 diffusion encoding direction schemes (6, 20, 20, 30, 60, respectively) using multiband (factor of two)^62^ EPI for accelerated acquisitions with anterior-posterior (AP) phase encoding direction. Three non-weighted diffusion images (b = 0 s/mm^2^) were also acquired. Other imaging parameters were: FOV = 220 mm × 220 mm, resolution = 2.5 × 2.5 × 2.5 mm^3^, matrix = 88 × 88, slices = 56, TR/TE = 4900/95 ms, bandwidth = 2104 Hz/pixel, a generalized autocalibrating partially parallel acquisitions (GRAPPA) factor of two. For geometric distortion correction, an additional image with b = 0 s/mm^2^ was acquired with the same imaging parameters, but with reversed (PA) phase encoding direction^63^. Standard clinical sequences including the MPRAGE (FOV = 256 mm × 256 mm, resolution = 1 × 1 × 1 mm^3^, matrix = 256 × 256, TR/TE/TI = 2100/3.19/900 ms), FLAIR (FOV = 256 mm × 256 mm, resolution = 0.7 × 0.7 × 5 mm^3^, matrix = 220 × 220, TR/TE/TI = 9000/90/2500 ms) and SWI (FOV = 256 mm × 256 mm, resolution = 0.7 × 0.7 × 3 mm^3^, matrix = 220 × 220) were done to assess the presence of any brain abnormalities.

### Working Memory Assessment

Working memory was assessed within one day that subjects underwent MRI scanning, using two WAIS-IV subtests^37^: 1) Digit Span (DS), which includes a) DS Forward (DSF) - examinees repeat back a sequence of numbers read to them, b) DS Backward (DSB) - examinees repeat back a sequence of numbers read to them, in reverse order, c) DS Sequencing (DSS) - examinees repeat back a sequence of numbers read to them, in ascending order, and 2) Letter-Number Sequencing (LNS) - examinees repeat back a sequence of numbers and letters read to them, numbers first in ascending order, followed by letters in alphabetical order. All measures were administered following standardized testing procedures, under the supervision of licensed clinical psychologists blinded to MRI results. Raw scores are converted to scaled scores (defined as mean of 10 with SD = 3, ranging 1–19) with higher scores indicating higher ability. In order to effectively eliminate age as a confounding factor for DS and LNS scores, age-corrected z-scores with a zero mean and a unitary variance were derived from the WAIS-IV normative sample (n = 2200) which was divided into 13 age bands spanning ages 16 to 90^37^. Both scaled scores and z-scores were used for analysis.

### Image Analyses

#### Diffusion image processing

The pre-processing steps for the diffusion weighted images include Marchenko-Pastur principal component analysis (MP-PCA) denoising^64^, Gibbs correction^65^, geometric EPI distortion correction (FSL’s function topup), eddy current distortion and motion correction (FSL’s function eddy), and outlier detection^66^. In-house image processing software developed in MATLAB R2017a (The Mathworks, Inc., Natick, MA) was used to calculate maps of WMTI metrics (AWF, ${\text{D}}_{\text{axon}}$, ${\text{D}}_{\text{e,}\Vert}$, ${\text{D}}_{\text{e,}\bot}$), as well as both DTI metrics (FA, mean diffusivity [MD], axial diffusivity [AD], radial diffusivity [RD]) and DKI metrics (MK, axial kurtosis [AK], RK).

#### Tract-based spatial statistics (TBSS)

Voxel-wise analyses were performed to reveal possible correlations between working memory subtest scores and the diffusion parametric maps by using the standard procedure of tract-based spatial statistics (TBSS)^67^. With TBSS, all subjects’ FA maps were registered to a FA template (http://fsl.fmrib.ox.ac.uk/fsl/fslwiki/FMRIB58_FA) and voxel-wise statistics were performed on FA values projected onto the study-specific WM skeleton by looking for maximum local values perpendicular to the skeleton. All other parametric maps underwent the same transformations and processes. The tract skeleton was thresholded at FA of 0.2 for DTI and DKI metrics and at FA of 0.4 to restrict analysis to WM regions consisting of single-fiber orientations for WMTI metrics, as recommended^25,57^. Statistical tests were conducted using the permutation-based nonparametric analysis routine ‘randomise’ with 10000 iterations; the design matrix was set up for the correlation analysis with covariates (https://fsl.fmrib.ox.ac.uk/fsl/fslwiki/GLM). The results were corrected for multiple comparisons using threshold free cluster enhancement (TFCE) in TBSS.

### Statistical Analysis

Spearman rank correlation was performed to assess the association of the DS and LNS test scores with age and length of education.

For TBSS analysis, age and sex were included as covariates. Statistical threshold level of p < 0.05 (corrected for multiple correction) was used. Spearman’s partial rank correlation coefficients were also calculated for regions on the skeleton with corrected p < 0.05, adjusted for age and sex using MATLAB R2017a.
